# Thermal properties of poly(N,N-dimethylaminoethyl methacrylate)

**DOI:** 10.1371/journal.pone.0217441

**Published:** 2019-06-05

**Authors:** Dawid Stawski, Aleksandra Nowak

**Affiliations:** Lodz University of Technology, Department of Material and Commodity Sciences and Textile Metrology, Division of Physical Chemistry of Polymers, Lodz, Poland; Duke University Marine Laboratory, UNITED STATES

## Abstract

Poly(N,N-dimethylaminoethyl methacrylate) (PDMAEMA) is a promising quite new polymer with very interesting properties. The thermal degradation process of PDMAEMA was investigated. The polymer was heated at specific time intervals, then heating was stopped, and infrared analysis was performed to obtain information on the structure of the solid residue. The thermal degradation process has a two-stage character. The limit temperature for the first decomposition step was about 390°C, after which the second stage of sample decomposition began. The order of disintegration of the macromolecules was determined. Activation energy values for the thermal decomposition process have been calculated; they are 89.8 kJ/mol for the first stage and 17.7 kJ/mol for the second stage of the degradation process.

## 1. Introduction

Bioactive polymers have recently attracted considerable attention in both academic and industrial research. The medical, food and textile industries are major end users of applied antimicrobials [1].

Poly(N,N-dimethylaminoethyl methacrylate) (PDMAEMA) is a polycation containing tertiary amine groups and possessing bioactive properties [2–5]. It is a weak polybase that has interesting temperature dependent solubility. PDMAEMA is a thermosensitive polymer that has a lower critical solution temperature (LCST), when the solid polymer is separated from the solution, because of hydrogen bond decomposition [6–8]. The LCST of PDMAEMA, which in aqueous solution at neutral pH is about 50°C, is higher than the human body temperature but could possibly be changed by its copolymerization. PDMAEMA has been used as a: (i) flocculant [9], (ii) carrier for drug delivery systems [10], (iii) ion exchange resin [11–12], (iv) mordant for ink printing [13], (v) membrane material for blood purification [14], (vi) cationic/anionic mosaic membrane for desalination [15], (vii) antimicrobial modifier for fibres [4–5], (viii) an independent textile flat material [16], and gel beads [17].

Thermogravimetric analysis (TG) is used to evaluate the thermal stability of different types of materials to find the maximum temperature at which the analysed polymer can be used. Additionally, important practical information can be obtained. With the use of a thermoanalyser, the relationship between sample mass loss and temperature can be obtained at a set heating rate, and one can determine temperature (T) or time (t) derivatives. In TG analysis, the sample can be (i) heated at a designated rate, (ii) observed at one temperature, or (iii) it can be analysed in a more complicated manner at a fixed temperature programme. The analysis results in a thermogram—a graph plotting sample weight (in mass units or per cent) against temperature (or time). The changes observed in sample mass can be caused by thermo-oxidation in an oxygen atmosphere or thermal degradation in an inert atmosphere.

PDMAEMA is a polymer that is important as a substance with high biological activity. The list of possible applications is long, and the number of possibilities will grow. So far, the thermal decomposition of this polymer has been analysed in nitrogen atmosphere in paper [18]. Polymer applications often require stability at higher temperatures in oxidizing conditions. PDMAEMA used as a textile modifier for medical applications must be resistant to sterilization, or the use of such materials as filters should take into account the changing external thermal conditions.

The following article makes up for this lack by presenting a full description of the PDMAEMA thermal degradation process. In this study, PDMAEMA was tested at thermo-oxidative conditions and changes in its structure were measured using FTIR spectroscopy.

## 2. Materials and methods

### 2.1. Materials

N,N-dimethylaminoethyl methacrylate (DMAEMA) (Sigma-Aldrich, Germany) was purified by distillation under vacuum (69–70°C, 1–2 mmHg).

PDMAEMA was prepared by radical polymerization of dimethylaminoethyl methacrylate initiated with azobisisobutyronitrile (AIBN, Merck, Germany). The purified DMAEMA monomer and AIBN initiator (0.4 g/l) were placed in a reactor made of polyethene terephthalate. The reactor was sealed and kept in an oven at 70°C for 1 week. After polymerization, the product was removed and cut into pieces for use. The molecular weight of the resulting polymer (Mn = 143.300 g/mol) was determined by gel permeation chromatography (GPC).

### 2.2. Methods

#### Fourier Transform Infrared Spectroscopy (FTIR)

The PDMAEMA was characterized using a Thermo Scientific Nicolet spectrophotometer with KBr (potassium bromide) pellets. The spectra were obtained from 4000–400 cm^-1^. Samples were analysed in the form of KBr pellet. Potassium bromide was previously dried (180 °C, 24h) to remove possible water content. Polymer samples were mixed with KBr and transmittance spectra were made (32 scans, resolution 4 cm^-1^). The second derivative was calculated by using the Perkin Elmer Spectrum 2000 programme.

#### Thermogravimetric analysis

The thermal analysis of all samples was carried out with a Perkin Elmer TGA 7 thermal analyser in a platinum measuring cell and using the Pyris program for data handling. The measurements were performed in air with a 10°C/min heating rate. The samples were heated up to 600°C, starting from room temperature. All measurements were repeated at least three times. Samples were acclimatized for at least one week in dry conditions (humidity below 5%) before measurement. Thermogravimetric analyzer is controlling sample weight before and during measurement.

## 3. Results and discussion

**Fig 1:**
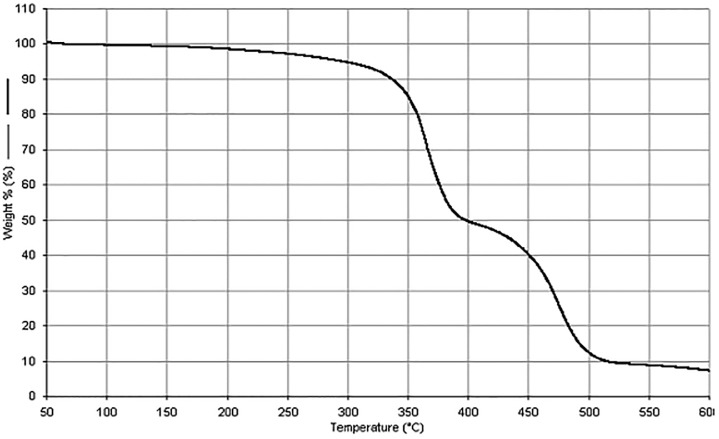
Thermogravimetric curve for a PDMAEMA sample heated in a 50–600°C range.

The thermal stability of PDMAEMA was investigated using TG analysis. Fig 1 shows the thermal degradation curve of the sample from room temperature to 600°C. This decomposition occurred in two steps. The first stage occurred in the 290–400°C range; and the second is located between 400–515°C.

In the next step, the obtained thermal degradation area was divided into temperature parts according to data presented in Table 1. The polymer was heated to an assumed temperature, and the measurements were stopped. Samples obtained at each stop (partially degraded) were analysed using FTIR spectroscopy.

**Table 1:** Temperatures of maximal heating for different PDMAEMA samples.

Sample number	Final temperature [°C]
1	280
2	315
3	350
4	385
5	420
6	455
7	490
8	525
9	560

**Fig 2:**
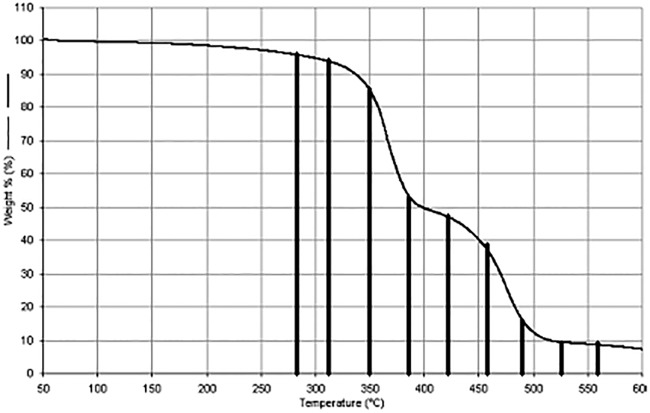
Thermal degradation area of PDAMEMA divided into analytical zones.

**Fig 3:**
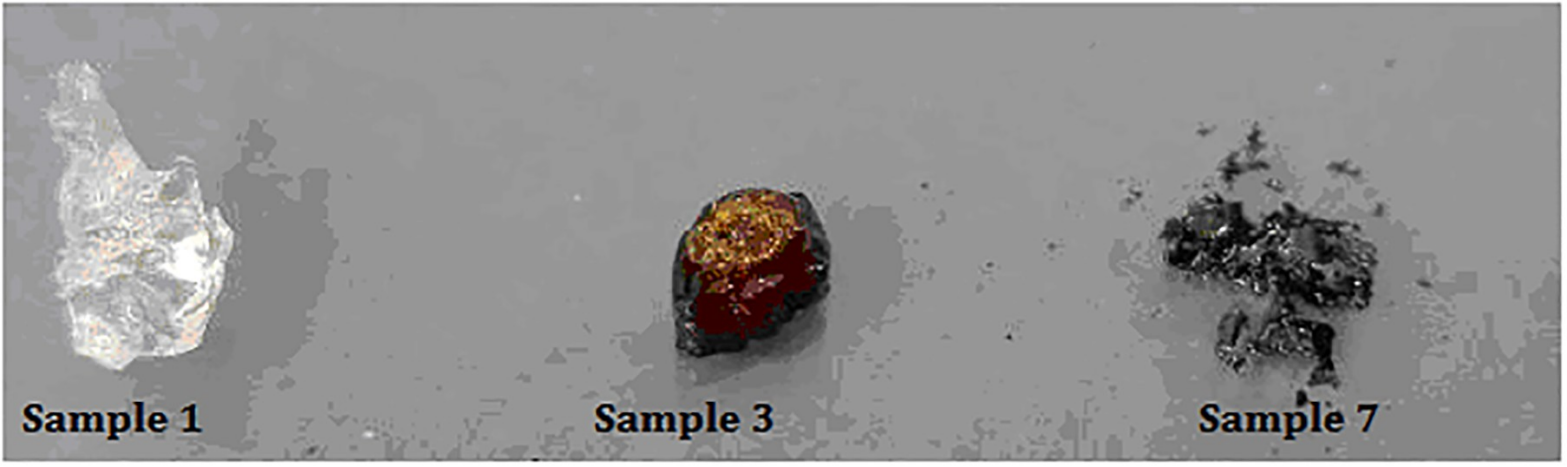
Visible effects of thermal decomposition of the PDMAEMA sample: Sample 1. before thermal treatment; Sample 3. sample heated to 350°C; Sample 7. sample heated to 490°C.

The thermal degradation of PDMAEMA divided into analytical parts, this effect is also presented more conclusively in Fig 2:

The largest single weight losses were observed in the 350–385 and 455–490°C ranges. After each stage of thermal decomposition, the sample was observed, recording its visible structure. As seen in Fig 3, the sample colour and structure changed; initially, it is a colourless, transparent, quite elastic material. Next, you can see a dark brown solid state (Sample 3) and finally black, fragile carbon residue (Sample 7).

**Fig 4:**
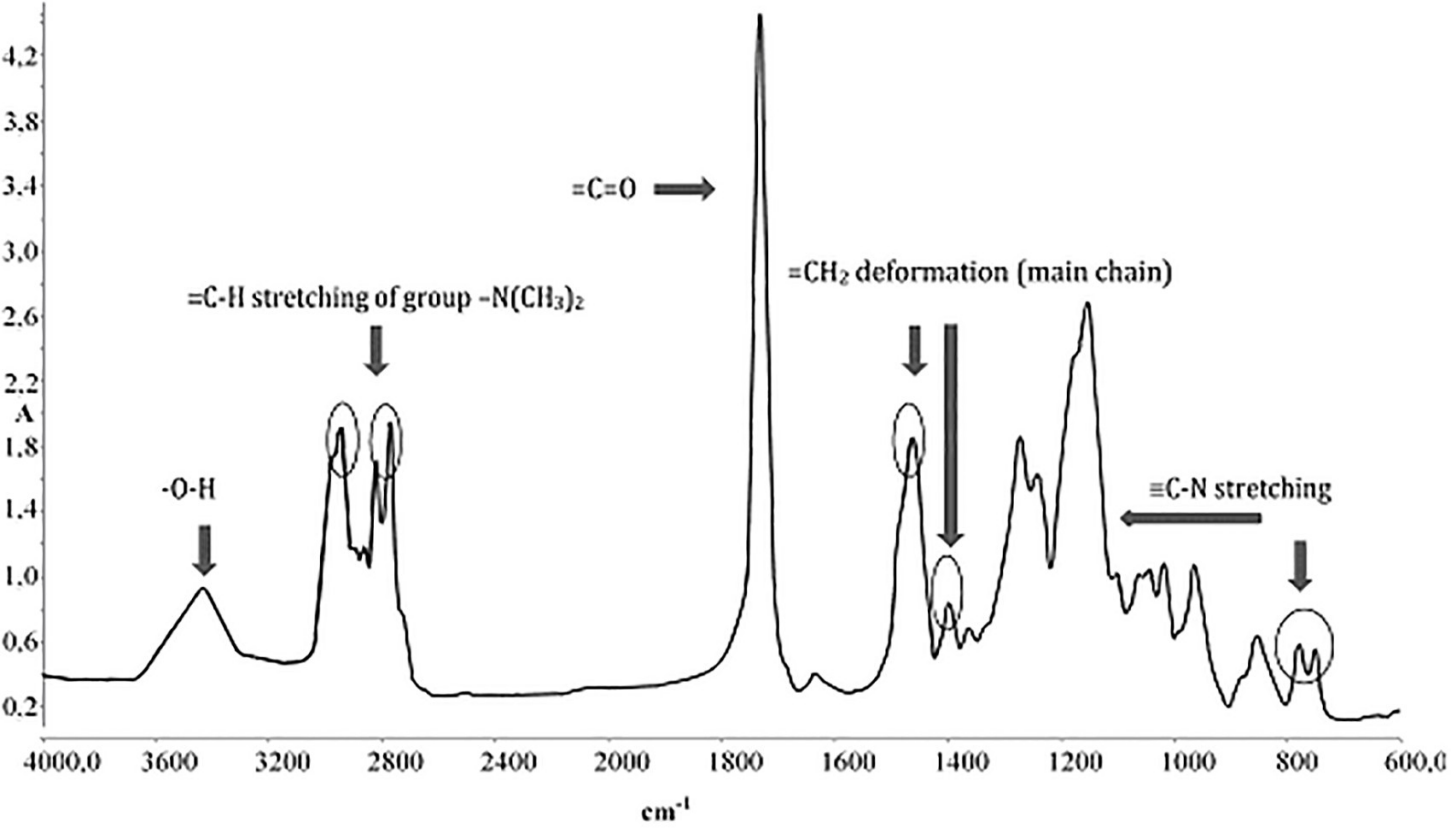
FTIR spectrum of an untreated PDAMEMA sample.

The basic tool, which was used for the analysis of solid thermal decomposition products was FTIR spectroscopy. An initial, unmodified sample was analysed. In Fig 4, you can see one of these spectra, with characteristic signals marked on it. The peak between 2700–300 cm^-1^ is connected with the C-H bond from −N(CH_3_)_2_ groups. The carbonyl signal from ester groups appears between 1600–1800 cm^-1^. Deformation vibrations from methylene groups on the main chain appear at 1400–1500 cm^-1^ and signals connected with C-N bond on the side chain are at 1150 and 750 cm^-1^. Due to high sample hydrophilicity, it is also possible to find the peak connected with −OH groups.

### 3.1 First thermal decomposition step

**Fig 5:**
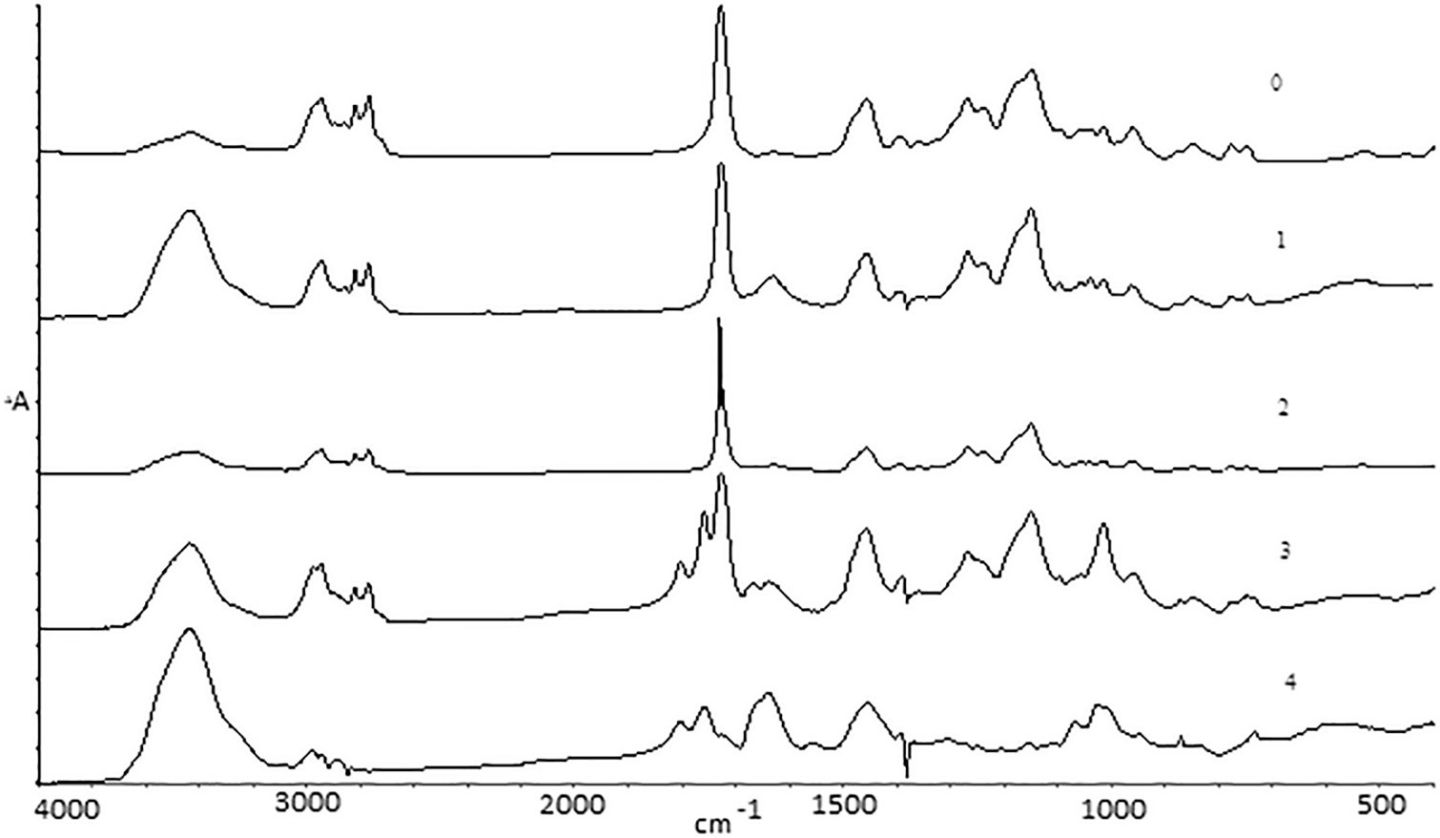
FTIR spectra of PDMAEMA samples (1–4) after thermal treatment in comparison with the untreated sample (0).

Thermal degradation of the sample occurs in two main steps. The first step takes place up to about 390°C, so it includes Samples 1–4. Fig 5 shows the spectra of these samples.

**Fig 6:**
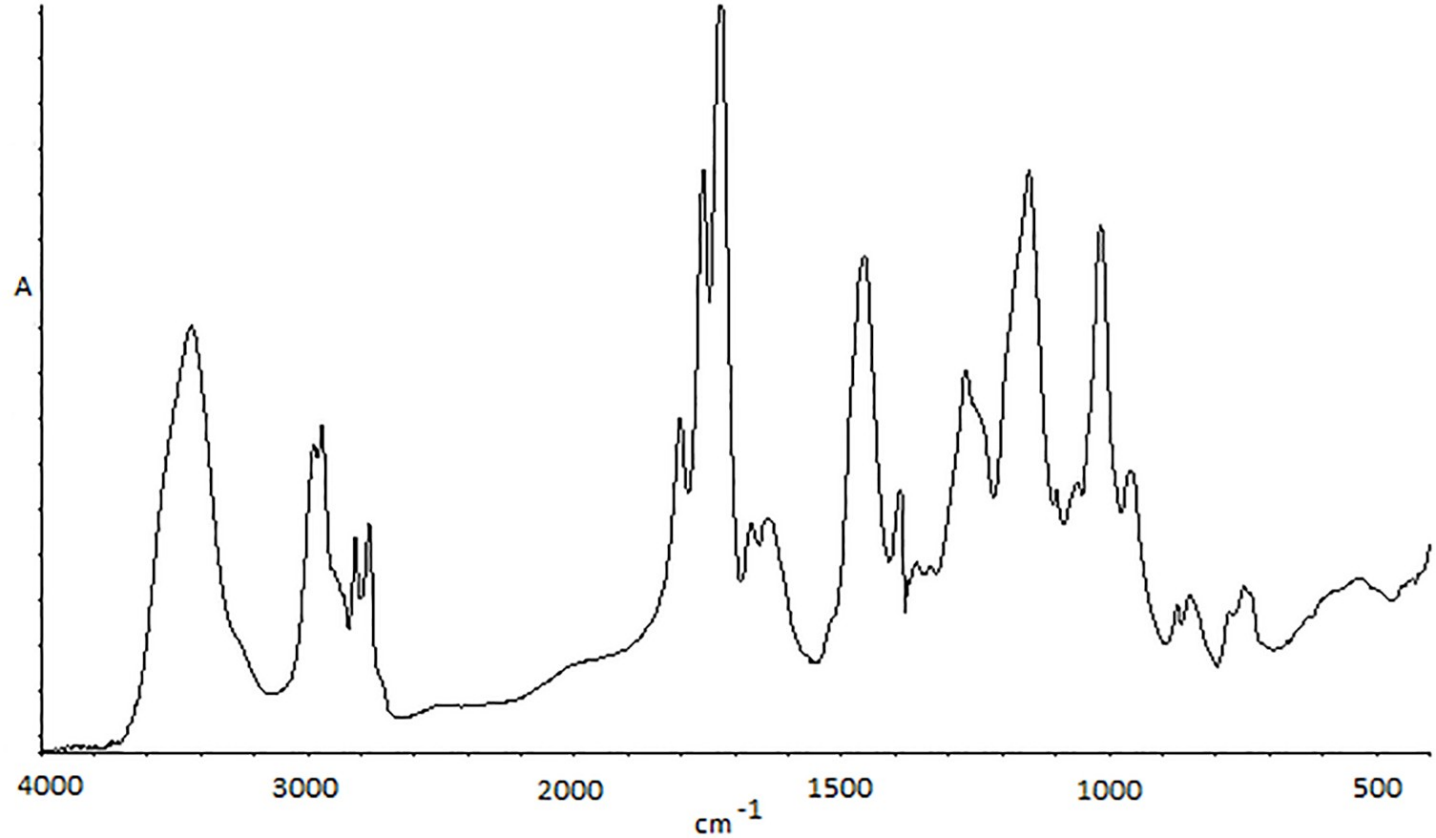
FTIR spectrum of sample 3.

The spectra for Samples 0–2 look similar, which means that thermal degradation starts at the 315–350°C range. New signals appear on thermogram for Sample 3. The peak in the 1600–1800 cm^-1^ area connected with carbonyl groups is divided into three signals—those peaks were identified as coming from esters (1728 cm^-1^), aldehydes (1761 cm^-1^) and carboxyl groups (1804 cm^-1^) (Fig 6). This relates to oxidation and volatile degradation of side groups.

**Fig 7:**
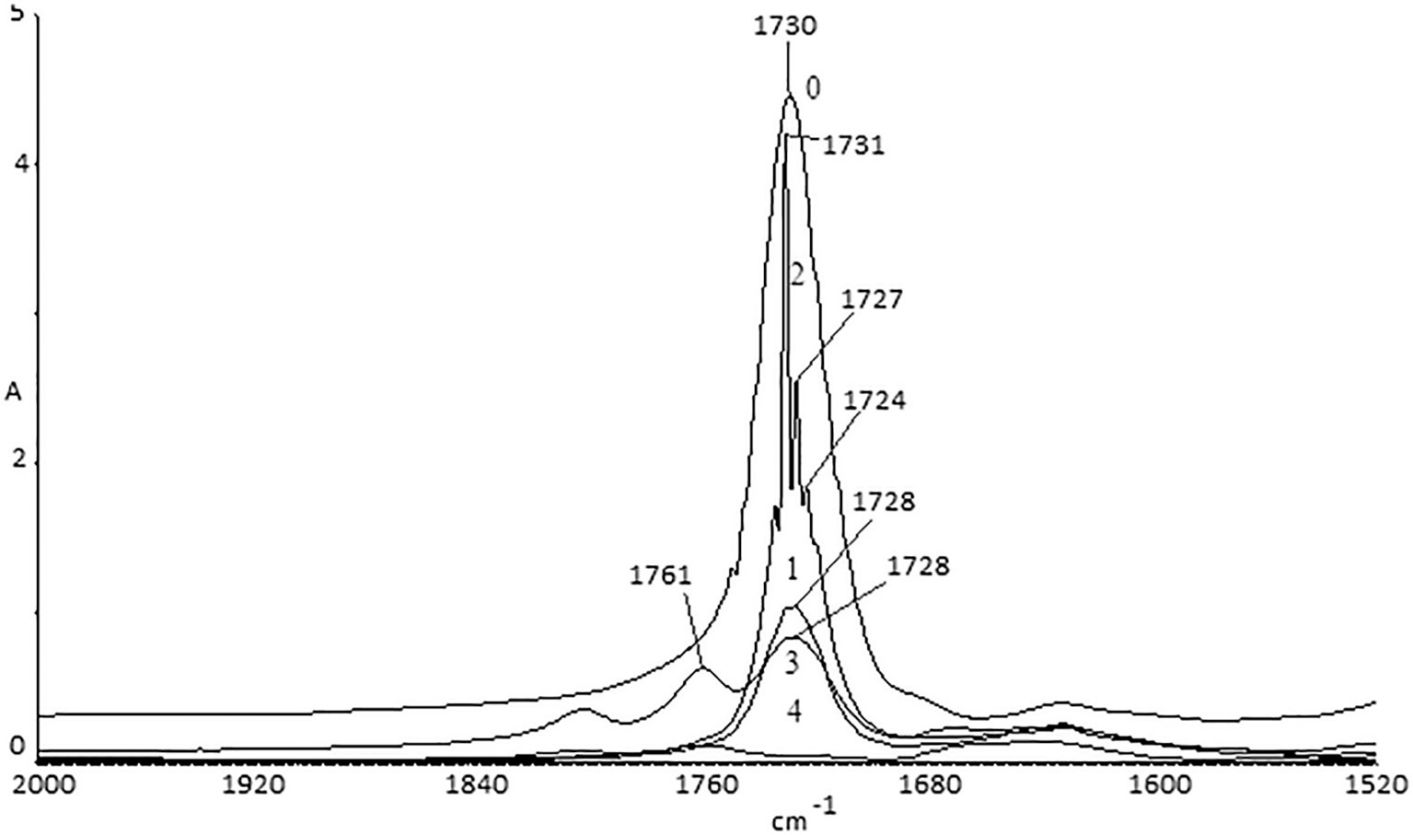
Comparison of the spectra for samples 0–4.

In Fig 7, one can see changes in the intensity of the signals caused by changes in carbonyl group character. Additionally, it is possible to see a peak with a maximum at 1671 cm^-1^, which is coming from amide groups.

**Fig 8:**
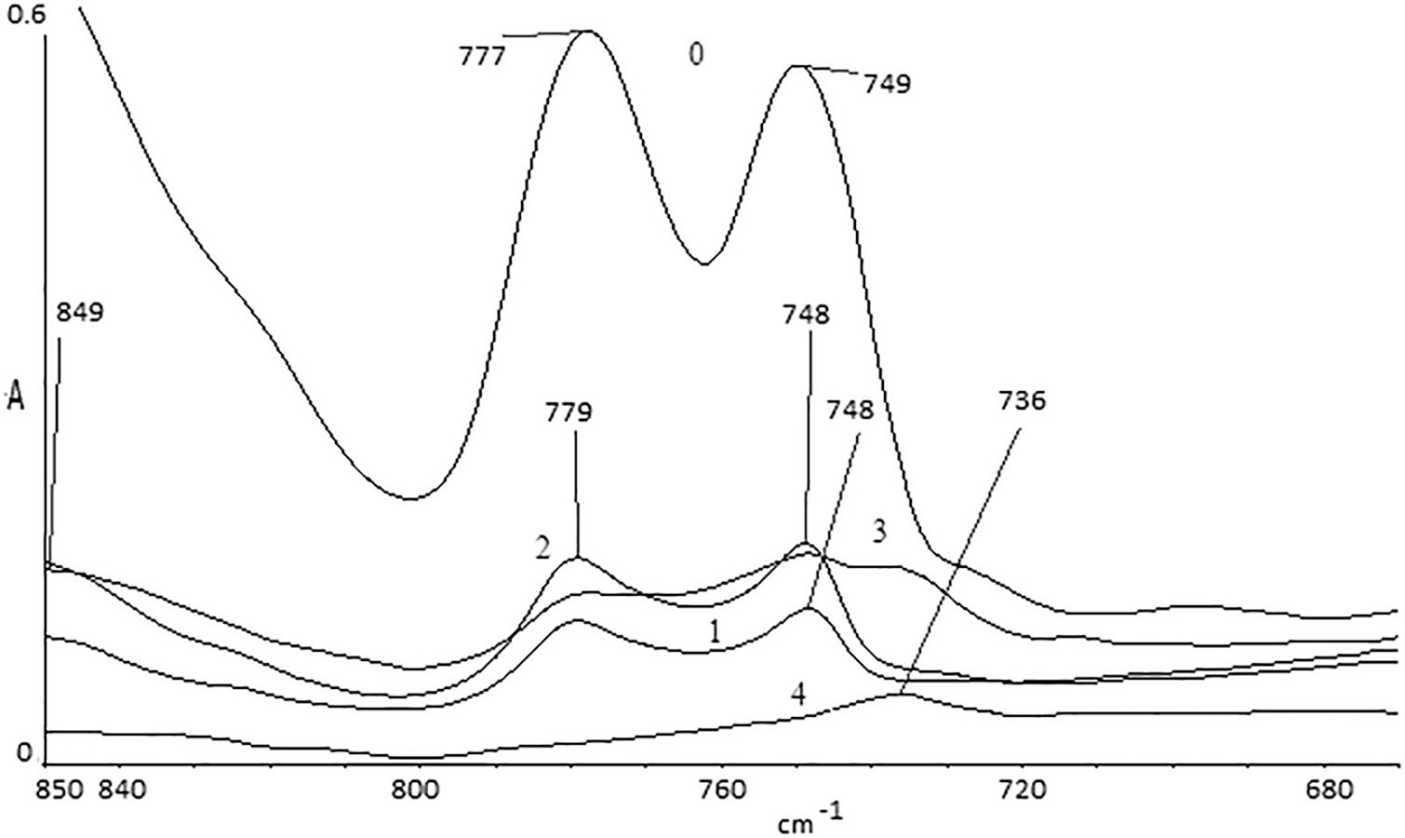
Comparison of the spectra for samples 0–4.

Localized signals coming from C-N groups were on the other part of the spectra (~ 800–730 cm^-1^). The decreasing intensity of that peak relates to the thermal decomposition of the sample–during degradation we can probably see emissions of volatile nitrogen oxides NO_x_ (Fig 8).

Changes in the proportion between signals coming from tertiary nitrogen (two maxima at ~780 cm^-1^ and ~750 cm^-1^) and a sum of peaks connected with different carbonyls (1800–1650 cm^-1^) are shown in Table 2. In Samples 0–2, one can find an interesting observation: The relationship between =N- / >CO decreases, which means that destruction of the carbon—nitrogen bond is faster than that of the carbonyl group. This can be clearly understood by considering the smaller energy of the C-N bond in comparison with C-C (Table 3). According to this observation, the fastest portion thermally decomposed is the part of the side group near tertiary nitrogen. A similar observation was made using the areas under the signals on the second derivative spectra (Table 2). Second derivative spectra were considered because quantitative analysis of this spectrum form improves the accuracy of the calculation by eliminating baseline shift and scattering in the spectra [19]. The derivative spectra can be use in quantitative determination by calibration using intensities of the bands in second derivative. The second derivative using proper algorithm would enhance the amplitude of the narrow band and suppress the broad band.

**Table 2:** Area under the peaks for samples 0–4 in the 1650–1800 and 730–800 cm^-1^ ranges.

Sample	Area [cm^-1^]		Areas proportion=N-/>CO zero order second order	
	>CO 1650–1800	=N- 730–800		
**0**	126.0	16.0	0.13	0.21
**1**	22.3	2.2	0.10	0.11
**2**	53.2	3.9	0.07	0.06
**3**	18.1	2.0	0.11	0.15
**4**	0.1	0.2	2.00	0

**Table 3:** Chemical bonds energies [20].

Bonds	Bond energy [kJ/mol]
C—N	305
H—C	415
C—C	347
C = O	741

The effect of destruction is clearly visible between Samples 3 and 4 (Fig 5). The spectrum for Sample 4 is very weak; the disappearance of signals indicates a slow loss of subsequent parts of the side groups.

### 3.2. Second thermal decomposition stage

**Fig 9:**
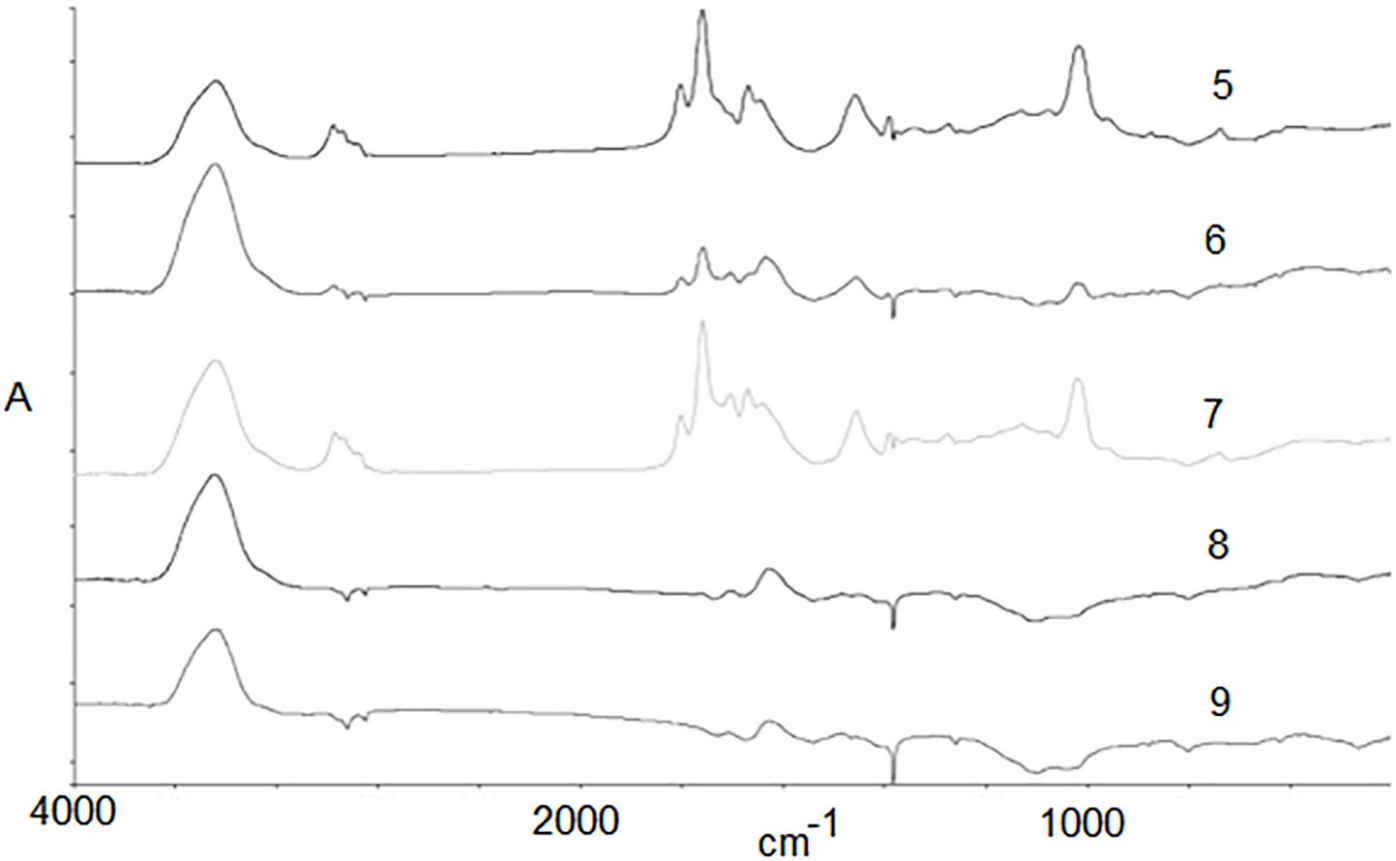
Full FTIR spectra for PDMAEMA heated at different temperatures: 5: 50–420°C; 6: 50–455°C; 7: 50–490°C; 8: 50–525°C; 9: 50–560°C.

The polymer exposed to thermal energy showed the characteristics of a two-stage decomposition. The beginning of the second phase of polymer degradation was observed at ~ 400°C. This disintegration is associated with Samples 5–9. Based on the thermogram (Fig 2) and spectral analysis (Fig 9), the thermal decomposition ends after exceeding ~525°C. Interpretation of signals indicates that, in the second stage of the decomposition, two processes overlap, thermal decomposition of side groups and initiation of the disintegration of the main chain.

**Fig 10:**
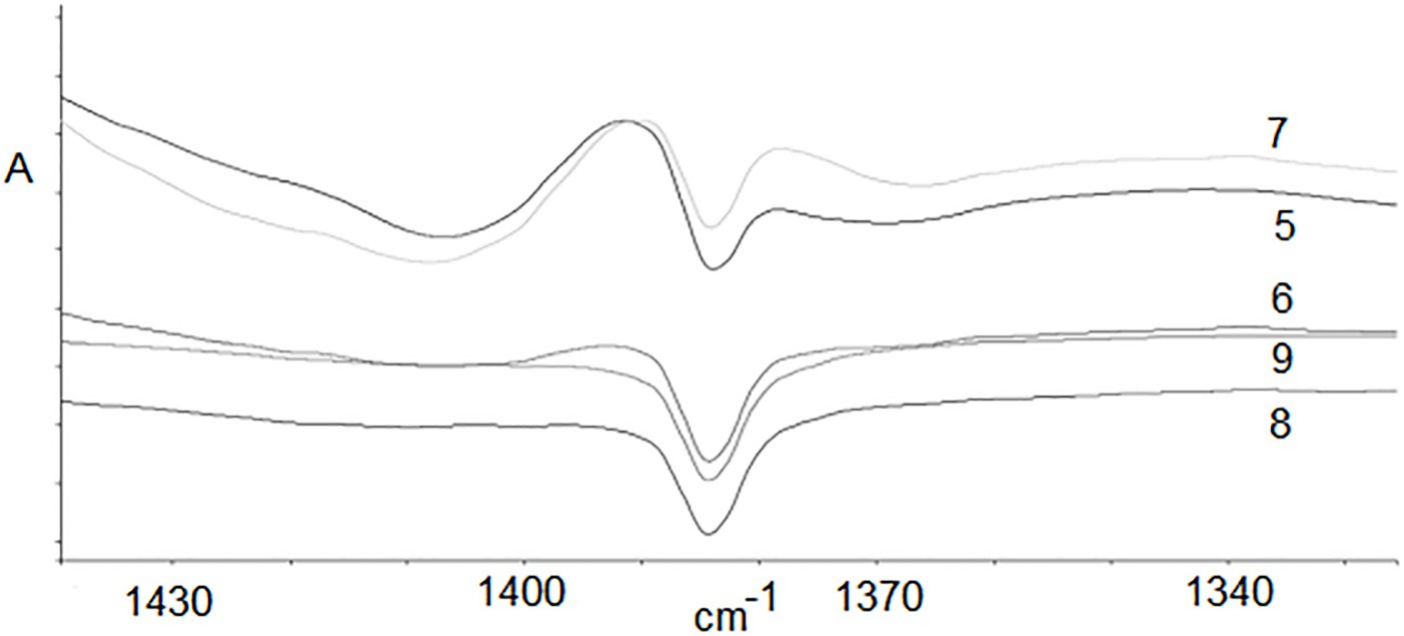
Part of FTIR spectra for samples 5–9 with signals coming from -CH_2_- groups in the main chain.

In Fig 10, the surface area under the signal coming from the -CH_2_- groups of the polymer backbone can be seen. The calculated areas (Table 4) indicate that a certain number of -CH_2_- groups are still present for Samples 5–7, while in later stages (Samples 8–9), we only deal with a solid residue after the thermal decomposition process.

Simultaneously with the disappearance of the last groups of the main chain, we can observe the destruction of left side groups (> CO).

**Table 4:** Area under peaks for samples 5–9 in the range 1650–1800 and 1390–1400 cm^-1^.

Sample	Area [cm^-1^]		Areas proportion-CH_2_-/>CO
	>CO 1650–1800	-CH_2_- 1390–1400	
**5**	3.60	0.13	0.04
**6**	1.10	0.05	0.05
**7**	12.60	0.10	0.01
**8**	0	0.04	0
**9**	0	0.03	0

In the last phase of the process, the main chain is disintegrated completely, resulting in unburned sample residue, i.e. ash.

### 3.3. Activation energy

There are many methods by which one can determine the kinetic parameters of thermal decomposition processes. They differ not only in the way of analysing the available data or the theoretical assumptions but also in the applied mathematical equations. The kinetics of changes occurring in the process of thermal degradation in synthetic air is described using the Coats-Redfern equation [21]:

$$log\frac{\alpha }{T^{2}}=log\frac{AR}{\beta E}(1-\frac{2RT}{E})-\frac{E}{2.3RT}$$ (1)

where: A—preexpotential factor [1/min]

E—activation energy [kJ/mol]

R—universal gas constant [kJ/molK]

T—temperature [K]

T_m_—temperature at maximum degradation velocity [K]

α—conversion rate

β—heating rate [K/min]

Plotting ln (α/T^2^) = f (1/T), E value can be calculated. It must be remembered that the equation is only true for zero reaction order, which results from the former simplifications. The results obtained by this method are true for low α, but they can be generalized for the whole of the process assuming that the reaction mechanism does not change during reaction duration. From the practical point of view this method is moderately laborious. It requires taking the α values from the thermogram and doing the necessary calculations for obtaining the plot.

The activation energy was calculated for both the first and second degradation step. The correlation coefficient, R^2^, was 0.93 for both phases of sample decomposition. By using the equation:

E=a*1000*8.3*8.31 (2)

where a is a constant for the linear equation for range 1 and range 2, it was possible to find E_A_ for both stages (Table 5).

**Table 5:** Activation energy for both degradation steps.

Stage of thermal degradation	Activation Energy [kJ/mol]
**I**	89.8
**II**	17.8

## 4. Conclusions

The purpose of this work was to study the thermal properties of poly(N,N-dimethylaminoethyl methacrylate). In the first stage of the work, the polymer was subjected to thermogravimetric analysis. Based on the thermogravimetric curve, it was found that the polymer degradation process was two-stage. The limit temperature for the first decomposition step was about 390°C, after which the second stage of sample decomposition began. To get complete information on the thermal decomposition of the sample, it was heated in various temperature ranges. Infrared analyses of samples obtained after each heating time indicate that decomposition of side groups was initiated in the first phase of thermal degradation, and the second stage combined processes of disintegration of side groups and the main chain.

Based on infrared spectra, with increasing thermal energy supplied, the intensity and proportions of the peaks changed. In addition to these phenomena, new signals appeared. They were identified when analysing Sample 3, which was heated in the 50–350°C range. As a result, the signal from the carbonyl group >CO was divided into four other peaks derived from the amide, ester, aldehyde and carboxyl groups. There was also a decrease in signal intensity for tertiary nitrogen (C-N). This means that in the first phase of the first stage of the thermal degradation, amine groups undergo the disappearance of side groups.

The second thermal decomposition step of PDMAEMA took place in the 390–560°C range. After identifying the FTIR spectra, Samples 5–7 continued to degrade the side polymer groups and initiated main chain degradation. In the case of Samples 8 and 9, whose maximum temperature limits were 525°C and 560°C, respectively, the polymer chain was completely disintegrated.

Activation energy values for the thermal decomposition process have been calculated; they are 89.8 kJ/mol for the first stage and 17.7 kJ/mol for the second stage of the degradation process.

## Supporting information

S1 File APDAMA 0.asc. (ASC)

S2 File APDAMA 1.asc. (ASC)

S3 File APDAMA 2.asc. (ASC)

S4 File APDAMA 3.asc. (ASC)

S5 File APDAMA 4.asc. (ASC)

S6 File APDAMA 5.asc. (ASC)

S7 File APDAMA 6.asc. (ASC)

S8 File APDAMA 7.asc. (ASC)
